# TG : HDL-C Ratio Is a Good Marker to Identify Children Affected by Obesity with Increased Cardiometabolic Risk and Insulin Resistance

**DOI:** 10.1155/2019/8586167

**Published:** 2019-12-01

**Authors:** Ahmad Kamil Nur Zati Iwani, Muhammad Yazid Jalaludin, Ruziana Mona Wan Mohd Zin, Md Zain Fuziah, Janet Yeow Hua Hong, Yahya Abqariyah, Abdul Halim Mokhtar, Wan Nazaimoon Wan Mohamud

**Affiliations:** ^1^Department of Pediatrics, Faculty of Medicine, University of Malaya, 50603 Kuala Lumpur, Malaysia; ^2^Endocrine and Metabolic Unit, Institute for Medical Research, Ministry of Health, Kuala Lumpur, Malaysia; ^3^Department of Pediatrics, Putrajaya Hospital, 62250 Putrajaya, Malaysia; ^4^Department of Social and Preventive Medicine, Faculty of Medicine, University of Malaya, 50603 Kuala Lumpur, Malaysia; ^5^Department of Sports Medicine, Faculty of Medicine, University of Malaya, 50603 Kuala Lumpur, Selangor, Malaysia

## Abstract

Metabolic syndrome (MetS) is an important predictor of cardiovascular diseases in adulthood. This study aims to examine the clinical utility of triglyceride to high-density lipoprotein ratio (TG : HDL-C) in identifying cardiometabolic risk and insulin resistance (IR) among children with obesity, in comparison with MetS as defined by the International Diabetes Federation (IDF). Data of 232 children with obesity aged 10–16 years were obtained from our study, MyBFF@school study, conducted between January and December 2014. Children were divided into tertiles of TG : HDL-C ratio. The minimum value of the highest tertile was 1.11. Thus, elevated TG : HDL-C ratio was defined as TG : HDL-C ≥1.11. Children with MetS were categorized based on the definition established by the IDF. Out of 232 children, 23 (9.9%) had MetS, out of which 5.6% were boys. Almost twofold of boys and girls had elevated TG : HDL-C ratio compared to MetS: 13.8% vs. 5.6% and 13.8% vs. 4.3%, respectively. Children with elevated TG : HDL-C ratio had lower fasting glucose compared to children with MetS (boys = 5.15 ± 0.4 vs. 6.34 ± 2.85 mmol/l, *p*=0.02; girls = 5.17 ± 0.28 vs. 6.8 ± 4.3 mmol/l, *p*=0.03). Additionally, boys with elevated TG : HDL-C ratio had a higher HDL-C level compared to those with MetS (1.08 ± 0.18 vs. 0.96 ± 0.1 mmol/l, *p*=0.03). There was no significant difference across other MetS-associated risk factors. Overall, TG : HDL-C ratio demonstrated higher sensitivity (42.7% vs. 12.9%) but lower specificity (74.8% vs. 93.2%) than MetS in identifying IR, either in HOMA-IR ≥2.6 for prepubertal children or HOMA-IR ≥4 for pubertal children. TG : HDL-C ratio in children with obesity is thus as useful as the diagnosis of MetS. It should be considered an additional component to MetS, especially as a surrogate marker for IR.

## 1. Introduction

Metabolic syndrome (MetS) is defined as the clustering of risk factors for cardiovascular diseases (CVDs) and type 2 diabetes (T2D), which include obesity, dyslipidemia, hypertension, and glucose intolerance [1]. With the increasing prevalence of overweight and obesity among children and youths, the “pediatric metabolic syndrome” has become a global public health concern. Children and adolescents with MetS are at greater risks of developing cardiovascular complications early, during the most productive years of their adult life [2].

Early identification and intervention are therefore crucial [3]. However, cutoffs and individual components used to diagnose MetS in children have not been standardized and need further elucidation. Among the most common definitions used for MetS is the one proposed by the International Diabetes Federation (IDF) [4], with fixed cutoffs for blood pressure, lipids, glycemia, and abdominal circumference points assessed by percentile. For children aged 10 years or older, the IDF proposed that the diagnosis of MetS should be based on waist circumference ≥90th percentile and the presence of two or more clinical features: elevated triglycerides, low HDL cholesterol, high blood pressure, or increased plasma glucose. Using this definition, a study involving 1014 Malaysian children aged 13 years showed that 258 (25.4%) were overweight/obese, out of which 10% had MetS [5].

Insulin resistance (IR) and central obesity are among the factors contributing to the anthropometric, physiological, and biochemical abnormalities in those with MetS [6, 7]. Nevertheless, due to the invasiveness and difficulty of measuring IR, the current definition of MetS in pediatric population regards elevated fasting glucose as a marker for glucose intolerance. Although the ability of the IDF criteria to predict CVD events has been established [8–10], the diagnosis of MetS is often made at the clinic or hospital level. Given the increasing burden of obesity among children, there is a need for an alternative: a simple method or tool with good sensitivity/specificity to identify children at risk of cardiometabolic diseases and IR in the community.

Current research has demonstrated the usefulness of triglyceride to high-density lipoprotein (TG : HDL-C) ratio in predicting cardiometabolic risk and IR [11–15]. However, evidence also indicated that there are ethnic variations [16, 17] and that genetic background is important in determining the presence of MetS among obese children [18]. Our study contributes to the existing literature by reporting the use of TG : HDL-C ratio in identifying cardiometabolic risk and IR among obese Malay school children, in comparison with MetS as defined by the IDF. In an earlier report, we showed that a significantly higher number of obese ethnic Malay school children at a higher tertile of TG : HDL-C ratio had acanthosis nigricans (AN) and MetS [11].

## 2. Materials and Methods

### 2.1. Study Design and Population

This study employed a cross-sectional design. Data were obtained from the MyBFF@school study conducted between January and December 2014 in Malaysia. MyBFF@school was a school-based lifestyle intervention program that included nutritional, physical activity, and psychology modules specifically designed for children with obesity. Detailed descriptions of the methodology have been previously published [11]. Ethical approval was granted by the Medical Research and Ethics Committee (MREC), Ministry of Health Malaysia. Written informed consent was obtained from parents or guardians, and every child was required to sign an assent form. All tests were performed in accordance with the approved guidelines.

### 2.2. Health and Physical Examination

Prior to health and physical examination, children were asked to fast overnight for at least 8 hours. Anthropometric measurements were performed by trained personnel, and health examinations were performed by medical officers and pediatricians. Standing height was measured without shoes to the nearest 0.1 cm using a calibrated stadiometer (Seca 217, Germany). Body weight and body fat mass were measured in light clothing without shoes and socks to the nearest 0.1 kg using a precalibrated body impedance analyzer (InBody 720, Korea). Waist circumference was measured two times to the nearest 0.1 cm over the skin midway between the tenth rib and the iliac crest at the end of normal expiration, using a nonextensible tape (Seca 201, Germany), and the mean was recorded. Two readings of blood pressure were measured after 5 minutes of rest using a mercury sphygmomanometer (Accoson, UK) in a seated position with the arm supported at the heart level, and the mean was recorded. Pubertal status was assessed (self-administered) using the Tanner staging scale [19, 20]. Children were also examined—by pediatricians—for the presence of acanthosis nigricans (AN) over the neck [21].

### 2.3. Biochemical Parameters

Venepuncture was performed by nurses and doctors. Blood samples were transported cold to the central laboratory at the Institute for Medical Research within two hours of collection and processed on the same day. Aliquots of serum/plasma samples were kept at −20°C prior to analysis. The HbA1c level was determined by cationic exchanged high-performance liquid chromatography (Adams A1c HA-8160, Arkray Inc., Japan) following the National Glycohemoglobin Standardization Programme guidelines. Fasting plasma glucose, triglycerides, total cholesterol, HDL, LDL, and liver enzymes (ALT, AST, and GGT) were analyzed using an automated analyzer (Dirui CS-400, China) with reagents purchased from Randox Laboratories (Antrim, UK), and the AST : ALT ratio was calculated. Fasting insulin concentration was measured using an automated enzyme immunoassay analyzer (TOSOH AIA-360, Japan). The interassay coefficient of variability (CV) for insulin at 8.7, 44.4, and 143.2 *μ*U/ml was 2.5%, 2.6%, and 2.4%, respectively.

### 2.4. Measures

Overweight and obesity were defined as the BMI *z*-score above 1 and 2 standard deviation, respectively, for age and sex, according to the WHO BMI chart (2008) [22]. Tanner staging was assessed by showing a standardized Tanner staging picture to the child. Stage 1 external genitalia development and breast development for boys and girls were classified as prepubertal, while stage 2 and above were defined as pubertal. AN was determined based on Burke's quantitative dichotomous score [21]. The IR status was based on the homeostasis model assessment (HOMA), calculated by multiplying the value of fasting plasma insulin and fasting plasma glucose and then dividing by 22.5 [21]. The pubertal transition from Tanner stage I to Tanner stage III or IV was associated with IR [23]. For prepubertal children, a score of HOMA ≥2.6 [24] was classified as IR, while a score less than 2.6 was classified as insulin sensitive. For pubertal children, a score of HOMA ≥4.0 was categorized as IR, while a score less than 4.0 was categorized as insulin sensitive [25]. MetS was established based on the definition proposed by the IDF [4]. It was considered present if the waist circumference measurement was ≥90th percentile of the Malaysian children waist circumference chart [26] with the presence of at least two of the following criteria: triglycerides ≥ 1.7 mmol/L, HDL cholesterol < 1.03 mmol/L, systolic blood pressure ≥ 130 mmHg and/or diastolic blood pressure ≥ 85 mmHg, or fasting plasma glucose ≥ 5.6 mmol/L [4]. The AST : ALT ratio less than 1 was categorized as a high risk of NAFLD, while a ratio more than 1 was categorized as a low risk of NAFLD [27].

## 3. Statistical Analysis

The normality test for continuous data was determined using the Kolmogorov–Smirnov test. Means and standard deviations (SDs) were calculated for continuous variables. Comparison of means between two groups was conducted using the independent-sample *t*-test, while categorical comparisons were made using the chi-square test. Statistical significance was set at 0.05. Sensitivity was calculated as the number of cases with elevated TG : HDL-C ratio or MetS who are IR by the HOMA-IR cutoff, divided by the total number of IR cases. Specificity was calculated as the total number of cases with normal TG : HDL-C ratio or absence of MetS that were insulin sensitive, divided by the total number of insulin-sensitive cases. Analyses were run using IBM Corp. Released 2013. IBM SPSS Statistics for Windows, Version 22.0. Armonk, NY: IBM Corp., and StataCorp. 2015. Stata Statistical Software: Release 14. College Station, TX: StataCorp LP.

## 4. Results

A total of 425 children with obesity (overweight/obese) participated in the MyBFF@school study, while 274 (65%) children consented for taking blood. Out of these 274 children, 232 were older than 10 years and had complete data of waist circumference and blood samples which were assayed for triglyceride, glucose, high-density lipoprotein (HDL-C), and liver aminotransferases (AST and ALT) and GGT.

Table 1 presents the characteristics of 232 children included in this study according to the sex group. Of 232 children, 23 (9.9%) were found to have MetS, out of whom 5.6% were boys. Pubertal status was comparable between girls and boys, despite the former being slightly older than the latter (13.3 ± 1.98 vs. 12.7 ± 2.0, *p*=0.002). More girls had AN than boys, but both groups had a similar IR status.  Table 2 describes the anthropometric and biochemical characteristics of study children. Even though boys showed higher BMI *z*-scores and larger waist circumference, girls had a higher percentage of body fat (BF) (42.5 ± 4.7 vs. 38.8 ± 6.7%, *p* < 0.001). There was no significant difference in other blood parameters except for liver enzymes. Boys had significantly higher AST, ALT, and GGT levels compared to girls.

**Figure 1:**
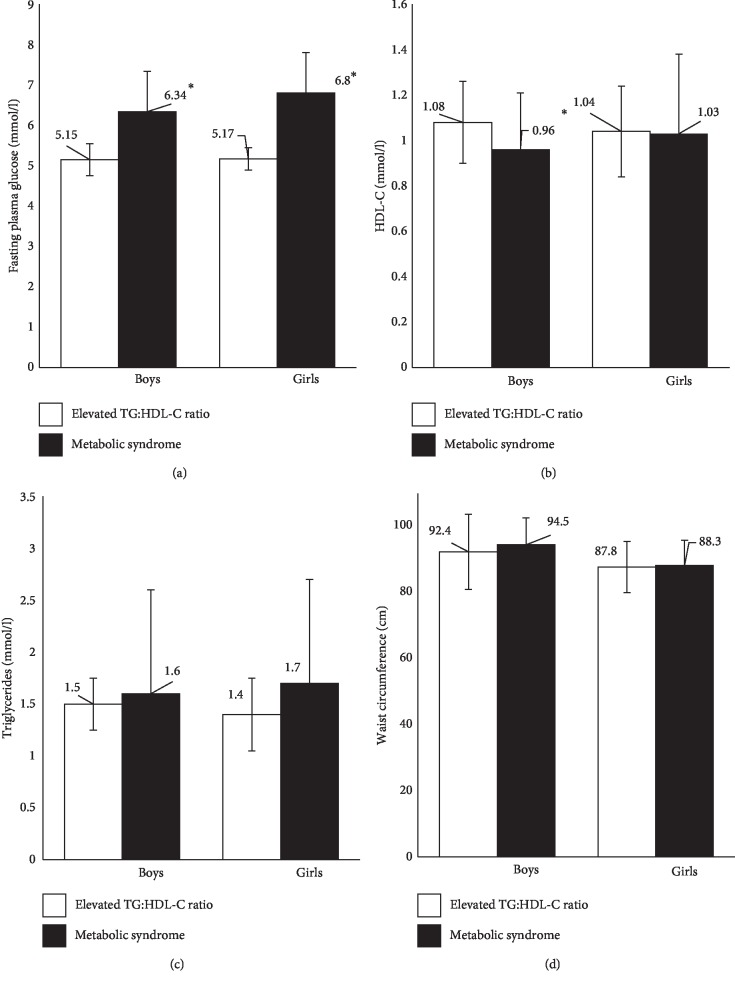
Comparison of (a) fasting plasma glucose, (b) HDL-C, (c) triglycerides, and (d) waist circumference between children with metabolic syndrome and elevated TG : HDL-C ratio. ^*∗*^*p* value < 0.005 by the independent-group *t*-test.

Children were divided into tertiles of TG : HDL-C ratio to determine the cut-off values separating the highest tertile from the other two lower tertiles. The minimum value of the highest tertile was 1.11 (Table 3).  Table 4 compares the MetS-associated risk factors for girls and boys, divided into those with elevated TG : HDL-C ratio (≥1.11) versus those with a diagnosis of MetS. It was shown that almost twofold of boys and girls had elevated TG : HDL-C ratio compared to MetS (boys = 13.8% vs. 5.6%, girls = 13.8% vs. 4.3%). Children with elevated TG : HDL-C ratio had lower fasting glucose compared to children with MetS (boys = 5.15 ± 0.4 vs. 6.34 ± 2.85, *p*=0.02; girls = 5.17 ± 0.28 vs. 6.8 ± 4.3 mmol/l, *p*=0.03) (Figure 1(a)). Additionally, boys with elevated TG : HDL-C ratio had a higher HDL-C level compared to those with MetS (1.08 ± 0.18 vs. 0.96 ± 0.1 mmol/l, *p*=0.03) (Figure 1(b)). There was no significant difference across other MetS-associated risk factors in either gender/group including the triglyceride level (Figure 1(c)), waist circumference (Figure 1(d)), and blood pressure.

The sensitivity and specificity of TG : HDL-C ratio and MetS in identifying children with either HOMA-IR ≥2.6 or HOMA-IR ≥4 are shown in Table 5. Overall, TG : HDL-C ratio showed higher sensitivity (42.7% vs. 12.9%) but lower specificity (74.8% vs. 93.2%) than MetS in identifying children with either HOMA-IR ≥2.6 or HOMA-IR ≥4.

## 5. Discussion

The utility of TG : HDL-C ratio in predicting IR among children with obesity has been reported in prior studies [11, 14, 28, 29]. Similarly, studies have also shown the use of TG : HDL-C ratio in identifying children at risk for MetS [14, 30, 31]. In this study, we found that TG : HDL-C ratio ≥1.11 (sensitivity = 42.7%, specificity = 74.8%) separated children with the highest tertile of TG : HDL-C ratio from the remaining two-thirds. To date, no specific TG : HDL-C cutoff has been established for children. We decided to use tertiles as they are a robust estimate and comparable to other studies [28, 30, 32]. Our cut-off value for TG : HDL-C ratio to identify MetS and IR among obese Malay school children is slightly lower than the cut-off value of 1.25 (sensitivity = 80%, specificity = 75%) established for obese Chinese children [30] and 2.0 (sensitivity = 55.6%, specificity = 72.9%) for overweight Korean children [28]. These differences could be attributed to ethnic and genetic variations, which have been said to influence the relationship between TG : HDL-C ratio and MetS across populations [16, 33]. For instance, Asians were reported to be more prone to abdominal obesity than Caucasians [34], so different cutoffs for TG : HDL-C ratio were used to identify IR and MetS.

We found no difference between elevated TG : HDL-C ratio and diagnosis of MetS with respect to all MetS risk markers, except for the glucose level. However, more children were identified at increased risks of cardiovascular diseases and T2D than did a diagnosis of MetS. In addition, we compared liver enzymes levels between those with elevated TG : HDL-C ratio and MetS, as elevated hepatic enzymes in adults were associated with obesity, IR, and T2D [35]. Like other risk factors, there was no difference in liver enzymes between the two groups. This indicates that TG : HDL-C ratio is as useful as the MetS diagnostic criterion in providing clinical information. Compared to MetS, TG : HDL-C ratio offered higher sensitivity (42.7% vs. 12.9%) but lower specificity (74.8% vs. 93.2%) in identifying children with IR among Malay children with obesity. Similar specificity (≈74%) but greater sensitivity (55.6%) of TG : HDL-C ratio were reported in a study of Korean children and adolescents with obesity [28]. However, the study used a lower value of HOMA-IR (≥3) and a higher TG : HDL-C ratio cutoff (≥2) to identify subjects with IR. Currently, there is no consensus on the HOMA-IR cut-off value which ranged between 1.14 and 5.56 [36, 37]. Despite the discrepancies of the HOMA-IR cutoffs, our findings suggested that TG : HDL-C ratio offers a slightly improved diagnostic value to screen for comorbidity, especially IR, among children with obesity. The International Society for Pediatric and Adolescent Diabetes (ISPAD) in its 2018 guidelines suggested that nonfasting lipid profiles are adequate for the screening of comorbidities among T2D pediatric population [38]. Thus, TG : HDL-C ratio can be an alternative, simple screening tool for comorbidities at the community or public health level.

We found that girls had higher body fat (BF) percentage despite showing lower BMI *z*-scores and smaller waist circumference compared to boys (Table 2). This is in line with an earlier study finding that the relationships between BMI and BF percentage were heterogeneous and varied between sexes and ethnic groups and across age groups [39]. There is a need, therefore, to reevaluate BF-based definition of obesity among the pediatric population. On the contrary, our results showed that boys had higher liver enzyme concentration than girls. This is consistent with the findings of Bussler et al. on the effects of gender on liver enzyme concentration [40] Differences between the two sexes are due to interactions between sex hormones and metabolic processes [41, 42], and it has been suggested that estrogen signaling in women protects against the development of IR and nonalcoholic fatty liver disease [43].

The application of TG : HDL-C ratio could be extended to distinguish metabolically healthy obese (MHO) individuals from unhealthy obese individuals. Despite meeting traditional BMI criteria for obesity, there appeared to be a normal/healthy metabolic profile that sets MHO individuals apart from their metabolically unhealthy obese counterparts [44]. To date, there is no universal definition for MHO, and research into the pediatric population is relatively lacking. However, for adults, MHO is most often defined using IR [45]. It was reported that, among other parameters (BMI, waist circumference, and apolipoprotein-B), MHO subjects showed lower TG : HDL-C ratio compared to those affected by obesity with cardiometabolic risk [46]. Another study showed that MHO individuals had lower levels of liver enzymes (such as ALT, AST, and GGT) and less fat in the liver [46]. Hence, TG : HDL-C ratio can be considered an additional marker to distinguish between MHO and metabolically unhealthy obese subjects. As shown in our study, those with elevated TG : HDL-C ratio had comparable risk to those with MetS. Having said this, the usefulness of TG : HDL-C ratio as a marker to distinguish between MHO and metabolically unhealthy obesity is beyond the scope of this paper.

Our study has a number of limitations: First, the prevalence of MetS reported might differ if a different definition of MetS was used. Our definition of MetS was based on the IDF, with fixed cutoffs for blood pressure, lipids, glucose, and abdominal circumference assessed by percentile and according to age groups. Although this is convenient, some children with MetS might have been excluded since the IDF requires central/abdominal obesity—measured by waist circumference—as a prerequisite for diagnosing MetS [47]. Second, our study subjects are restricted to ethnic Malay children. This does not represent the true multiracial population in Malaysia. It has long been established that the predictability of TG : HDL-C ratio as a surrogate marker for IR is influenced by ethnic and genetic variations [16, 33]. Our future studies thus will attempt to address this limitation—by obtaining adequate representation of all ethnic groups—in order to evaluate the use of TG : HDL-C ratio as a measure of IR among Malaysian children.

## 6. Conclusion

In conclusion, the determination of TG : HDL-C ratio among obese Malay children provided equally useful clinical information to MetS. TG : HDL-C ratio should be considered an additional component to MetS, as a surrogate marker for IR. Additionally, the advantage of TG : HDL-C ratio is its routine measurement in children with obesity that can be done in a nonfasting state and conveniently measured using a portable analyzer with good precision at low cost. Given that existing studies on TG : HDL-C ratio are mostly cross-sectional in nature, there is a need to carry out longitudinal studies across different ethnic groups to evaluate the utility of TG : HDL-C ratio in identifying those at risk for T2D and CVDs.

## Figures and Tables

**Table 1:** Clinical characteristics of 232 children by gender.

	Boys (*n* = 114, 49.2%)	Girls (*n* = 118, 50.8%)	*X* ^2^	*p* value	All
Age (mean ± SD)	12.7 ± 2.0	13.3 ± 1.98	NA	0.02^a^	12.6 ± 2.01
Pubertal status					
Prepubertal	42 (37.2)	29 (25.4)	3.632	0.07	71 (31.3)
Pubertal (Tanner stage ≥ 2)	71 (62.8)	85 (74.6)			156 (68.7)
Abdominal obesity					
WC < 90^th^ centile	11 (9.6)	15 (12.7)	0.547	0.46	26 (11.2)
WC ≥ 90^th^ centile	103 (90.4)	103 (87.3)			206 (88.8)
BMI *z*-score >1 or 2 SD					
Overweight	17 (14.9)	33 (28)	5.884	0.02	50 (21.6)
Obese	97 (85.1)	85 (72)			182 (78.4)
Acanthosis nigricans					
Presence	47 (42)	74 (65.5)	12.52	<0.001	121 (53.8)
Absence	65 (58)	39 (34.5)			104 (46.2)
Insulin resistance					
Prepubertal					
HOMA-IR < 2.6	15 (35.7)	9 (31)	0.168	0.68	24 (33.8)
HOMA-IR ≥ 2.6	27 (64.3)	20 (68.9)			47 (66.2)
Pubertal					
HOMA-IR < 4	35 (49.2)	44 (54.3)	0.094	0.76	79 (50.6)
HOMA-IR ≥ 4	36 (50.8)	41 (45.7)			77 (49.4)
Liver enzyme test					
Low risk (ALT : AST >1)	97 (85.1)	104 (88.1)	0.465	0.495	201 (86.6)
High risk (ALT : AST≤1)	17 (14.9)	14 (11.9)			31 (13.4)
Metabolic syndrome					
Nonmetabolic syndrome	101 (88.6)	108 (91.5)	0.557	0.455	209 (90.1)
With metabolic syndrome	13 (11.4)	10 (8.5)			23 (9.9)

^a^Independent-group *t*-test; NA: not available.

**Table 2:** Anthropometric and biochemical characteristics of 232 children.

Cardiovascular risk factors	Boys (*n* = 114)	Girls (*n* = 118)	*p* value	All
Obesity				
Mean BMI *z*-score	2.8 ± 0.9	2.4 ± 0.7	<0.001	1.04 ± 0.82
Mean waist circumference (cm)	91.1 ± 10.9	85.6 ± 8.3	<0.001	88.34 ± 10.1
Mean body fat (%)	38.8 ± 6.7	42.5 ± 4.7	<0.001	40.7 ± 6
Blood lipids				
Mean total cholesterol (mmol/l)	4.62 ± 0.76	4.56 ± 0.8	0.6	4.59 ± 0.79
Mean TG (mmol/l)	1.1 ± 0.5	1.1 ± 0.5	0.23	1.1 ± 0.46
Mean HDL-C (mmol/l)	1.1 ± 0.2	1.17 ± 0.2	0.32	1.2 ± 0.2
Mean LDL-C (mmol/l)	3.2 ± 0.86	3.2 ± 0.82	0.9	3.2 ± 0.84
Blood pressure				
Mean systolic blood pressure (mmHg)	111 ± 11	108 ± 10	0.11	110 ± 10
Mean diastolic blood pressure (mmHg)	73 ± 9	69 ± 9	0.01	70 ± 9
Insulin resistance markers				
Mean TG : HDL-C	0.95 ± 0.47	1.04 ± 0.53	0.16	0.99 ± 0.5
Mean fasting glucose (mmol/l)	5.4 ± 1.35	5.4 ± 1.1	0.23	5.39 ± 1.22
Mean insulin	18.4 ± 12.2	21.59 ± 26.8	0.243	
Liver enzymes/NAFLD blood markers				
Mean ALT (U/l)	19.1 ± 18.1	13.3 ± 11.7	0.004	16.1 ± 15
Mean AST (U/l)	27.6 ± 21.67	20.06 ± 12.55	0.001	23.7 ± 18
Mean GGT (U/l)	31.9 ± 34.35	20.8 ± 13.6	0.001	26.2 ± 26.5
Mean AST : ALT	2.07 ± 1.9	1.89 ± 1.8	0.447	1.9 ± 1.8

*p* value was obtained from comparison of means using the independent-sample *t*-test.

**Table 3:** Minimum, mean, and maximum of the plasma TG : HDL-C concentration ratio tertiles by gender.

	Boys	Girls	All
Tertiles 1	(0.33, 0.57, 0.7)	(0.27, 0.55.0.71)	(0.27, 0.56, 0.71)
Tertiles 2	(0.72, 0.9, 1.1)	(0.71, 0.88, 1.1)	(0.71, 0.89, 1.1)
Tertiles 3	(1.11, 1.58, 3.21)	(1.11, 1.48, 2.75)	(1.11, 1.54, 3.21)

**Table 4:** Comparison of metabolic syndrome-associated risk factors between children with metabolic syndrome (MetS) and elevated TG : HDL-C ratio (TG : HDL-C ≥ 1.11).

Biochemical/clinical parameters	Boys (*n* = 45)			Girls (*n* = 42)		
	Elevated TG : HDL-C ≥1.11 (*n* = 32, 71.1%)	MetS (*n* = 13, 28.9%)	*p* value	TG : HDL-C ≥1.11 (*n* = 32, 75%)	MetS (*n* = 10, 25%)	*p* value
Age	12.6 ± 1.97	13.1 ± 1.26	0.482	12.6 ± 1.9	13.2 ± 1.5	0.46
*Cardiovascular risk factors*						
Obesity						
Mean BMI *z*-score	2.9 ± 0.9	2.88 ± 0.6	0.95	2.6 ± 0.7	2.5 ± 0.5	0.78
Mean body fat (%)	38.5 ± 7.3	39.7 ± 4.9	0.623	42.6 ± 4.9	43.9 ± 3.7	0.337
Blood lipids						
Mean total cholesterol (mmol/l)	4.68 ± 0.78	4.37 ± 0.9	0.253	4.5 ± 0.91	4.67 ± 0.9	0.623
Mean LDL-C (mmol/l)	3.33 ± 0.84	3.2 ± 1.14	0.636	3.2 ± 0.98	3.4 ± 0.74	0.439
Blood pressure						
Mean systolic blood pressure (mmHg)	112 ± 9	116 ± 11	0.26	108 ± 12	111 ± 9	0.476
Mean diastolic blood pressure (mmHg)	73 ± 8	75 ± 8	0.468	69 ± 10	70 ± 7	0.885
*Insulin resistance markers*						
Mean TG : HDL-C	1.44 ± 0.33	1.69 ± 0.83	0.17	1.34 ± 0.25	1.66 ± 0.77	0.045
Mean insulin	21.68 ± 13.44	22.54 ± 22.54	0.844	20.6 ± 11.9	26.7 ± 23.27	0.28
*Liver enzymes/NAFLD blood markers*						
Mean ALT (U/l)	22.6 ± 17.3	19.23 ± 19.4	0.57	14.8 ± 10.9	22.2 ± 27.2	0.229
Mean AST (U/l)	32 ± 28.24	27.7 ± 24.76	0.634	23.3 ± 16.4	25.1 ± 21.6	0.80
Mean GGT (U/l)	34.0 ± 25.3	43.23 ± 64.9	0.507	21.2 ± 13.2	30.7 ± 19.9	0.09
Mean AST : ALT	1.64 ± 1.1	1.64 ± 0.62	0.98	2.36 ± 3.14	1.7 ± 1.1	0.64

*p* value was obtained from comparison of means using the independent-group *t*-test; NA: not available.

**Table 5:** % sensitivity (true positives/all positives) and specificity (true negatives/all negatives) of HOMA-IR ≥4 (pubertal) and HOMA-IR ≥ 2.6 (prepubertal) for TG : HDL-C ratio ≥ 1.11 and metabolic syndrome.

	TG : HDL-C ratio ≥ 1.11		Metabolic syndrome	
	Sensitivity	Specificity	Sensitivity	Specificity
HOMA-IR ≥2.6 or≥4	42.7 (53/124)	74.8 (69/103)	12.9 (16/124)	92 (96/103)

## Data Availability

The datasets used and/or analyzed during the current study are available from the corresponding author on reasonable request.
